# Enhanced soil attributes, yield and fruit quality of Medjool date palm (*Phoenix dactylifera* L.) in response to filter mud/cake for drylands sustainability

**DOI:** 10.1371/journal.pone.0343806

**Published:** 2026-03-02

**Authors:** Atef Abo-Ogiala, Abdel-Moety Salama, Mohamed S. Aboryia, Ahmed M. Fikry, Nazih Y. Rebouh, Mohamed S. Shokr, Naglaa Khalafallah

**Affiliations:** 1 Horticulture Department, Faculty of Agriculture, Tanta University, Tanta, Egypt,; 2 Horticulture Department, Faculty of Agriculture, Kafrelshaikh University, Kafr El-Shaikh, Egypt; 3 Horticulture Department (Pomology), Faculty of Agriculture, Damietta University, New Damietta, Egypt; 4 Horticulture department, Faculty of agriculture, Zagazig university, Zagazig, Egypt; 5 Institute of Environmental Engineering, RUDN University, Moscow, Russia; 6 Soil and Water Department, Faculty of Agriculture, Tanta University, Tanta, Egypt; Sultan Qaboos University College of Science, OMAN

## Abstract

Soils of drylands in arid and semiarid conditions need developing approaches to improve the holding capacity of water and nutrients to avoid desertification. In this study, we examine the characteristics of filter mud/cake under the intended conditions and verify the additional value of employing it as a byproduct of the sugarcane industry, avoiding its negative impacts on the environment. This was accomplished in two prosperous seasons in 2022 and 2023 by using uniform Medjool date palms (*Phoenix dactylifera* L.) that were ancient, vigorous, fruiting, and healthy at a private farm in Dakhla Oasis, New Valley Governorate, Egypt. The application of filter mud as a soil amendment significantly led to better results in all parameters under investigation, i.e., soil attributes, yield parameters, and fruit quality, compared to the additive of animal and poultry manure used separately. However, the results suggested applying filter mud mixed to poultry manure (T6), that showed better results compared to other treatments, i.e., water content at field capacity (WFC) were 12.58 and 12.59%, whereas NPK content in soil were 35.23 and 35.25 mg/kg N, 5.33 and 5.37 mg/kg P, 61.21 and 61.18 mg/kg K in both seasons 2022 and 2023, respectively. Moreover, T6 treatment showed the best results of yield per date palm tree, i.e., 65 and 66 Kg with the highest fruit quality indicators, i.e., 8 and 8.1 cm fruit length, 2.7 and 2.8 cm fruit diameter in both seasons as respectively. The study presented filter mud as promising application for soil amendment in arid and semiarid conditions with direct contribution to key Sustainable Development Goals (SDGs). Subsequently, enhancing soil health and sustainable agriculture (SDG 2: Zero Hunger), fostering green innovation in by-product valorization (SDG 9: Industry, Innovation, and Infrastructure), and promoting the sustainable management and reduction of industrial waste (SDG 12: Responsible Consumption and Production).

## 1. Introduction

The importance of using dates in food is highlighted by the cultivated area of date palm globally, which reached around 1.381 million hectares in 2023 [1]. The Middle East region alone represented 57% of the total area, especially in Egypt, Iran, Saudi Arabia, and the United Arab Emirates, giving almost 9.66 million metric tons of dates in 2021 [2,3]. In Egypt, the total cultivated area reached 56332.92 hectares distributed in two main regions: Nile valley lands around 41672.82 hectares and 14660.10 hectares were cultivated in new reclamation desert lands with the total yield production of 1710603 tons in 2021 [2,3]. The Medjool cultivar in Egypt has recently proved to be the best adaptation for dry and semi-dry conditions with the optimal growth, yield and fruit quality, subsequently increasing in many desert regions, especially in Upper Egypt including New Vally governorate [1].

To establish a connection with the previous information and align with the target of that work it was necessary to consider the sugarcane as a common crop in Upper Egypt from ancient time which leaves huge residuals after harvest, subsequently causing some negative impacts on the surrounding environment. The cane crop is spread across in five Governorates, i.e., El Menia; Sohag; Qena; Luxor and Aswan with an annual production yield of 16 million tons [4]. Mass of filter mud/cake, which is a final residue of sugar manufacturing, is accumulated in an open storage area for a couple of months, passes to natural heat treatments and subsequent fermentation until the drying stage, and changes to a dark brown color. Therefore, it was necessary to find a friendly method for recycling the filter mud to avoid such as accumulation and subsequently avoid any negative impacts on the environment. Such demand encouraged us to use the filter mud as organic matter in comparison with the common ones, such animal and poultry manure. The recent description of filter mud was introduced by Alabi et al., [5] a rich source of fibers substrates, especially with cellulose and lignin, in addition to other molecules such as nitrogen, inorganic salts, protein, cane wax, and coagulated colloids that look like soil clay. Filter mud showed a soft texture, spongy shape, and amorphous appearance. In Egypt, the first academic work on filter mud as compost and organic fertilizer was described in detail by Nakhla et al., [6]. Abdel-Galeil et al., [7] recommended using filter mud as an organic matter source instead of compost for preparing growth media for date palm cv. For Sakkoty plantlets at the adaptation stage at the end of the tissue culture reproduction. On the other hand, Noufal [8] highlighted the positive effects of filter mud on improving desertic soil physicochemical and microbial properties. The media containing filter mud resulted in better growth in comparison to other media, such as animal or poultry manure, which are good sources as compost for newly cultivated soil and for organic farming. However, they can introduce weed seeds and the pathogen sources, especially upon the absence of heat treatments [9,10]. Ait-El-Mokhtar et al., [11], reported that compost mitigates salt-induced effects on growth, nutritional, physiological and biochemical responses of date palm. Moreover, Luan et al., [12] explored the benefits of manure as chemical fertilizers on the development soil community diversity. This means that organic matter as a substrate in general alleviates salt stress and lowers accumulation of lipid peroxidation and H_2_O_2_, while increasing accumulation of soluble sugars and proline. El Janati et. al., [13] and Karboutet al., [14] stated that the addition of organic matter to date palm orchards enhanced soil structure, subsequently increasing the water–and nutrient-holding capacity. However, according to Rashwan et al., [15] poultry manure easily losses its held nutrients, making it necessary to mix it with biochar to prolong its benefits for soil amendment and as a fertilizer. Closer to our target of this study, Alabi, et al., [5] revealed that the application of filter mud reduces the cost of chemical fertilizer by 15%−20%. In addition, Morales-Maza et al., [16] found that compost application required chemical fertilization to improve soil properties, which led to enhanced yield and fruit quality compared to untreated palms. The aims of this work were to contribute to SDG targets by exploring new agricultural practices that advance sustainability in clean agriculture production as follow: -

1. Explore the additive value of filter mud as compost for new reclaimed soils, especially in drylands which had not been previously investigated in comparison to animal and poultry manure, subsequently avoiding negative impacts on environment due to its accumulation.
2. Highlight the best treatment that brings improving soil attributes, yield and fruit quality of Medjool date palm (*Phoenix dactylifera* L.)

## 2. Material and methods

### 2.1. Experimental design

**Fig 1:**
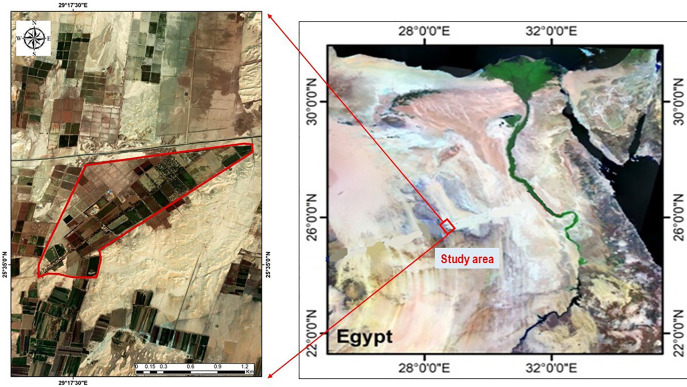
Location Map of the study area.

The Medjool date palm trees used in this experiment were grown in a private orchard at coordinates 29º 18′ 08.5ʺ E and 25 º 35 ′ 43.2ʺ N in Bashandi Village, Dakhla Oasis, New Valley Governorate, Egypt (Fig 1) (https://earthexplorer.usgs.gov/ accessed on 10/2025). No special permits were required for this study, as the research was conducted in a publicly accessible area and did not involve interaction with protected or endangered species or disturbance to regulated ecosystems.

The area is incredibly dry because it is a portion of the hot, dry Sahara, where descending air from the equatorial region creates the tropical continental zone, a stable hot air mass [17]. According to Dakhla’s climate parameters, the area is extremely dry. The typical yearly temperature is between 12 °C in January and 31 °C in July. In January and June, evapotranspiration rates can reach 2.6 mm/day and 9.1 mm/day, respectively. Rainy months are January and February, and annual precipitation does not surpass 1 mm. Wind speeds vary from 3 knots in December to 5.5 knots in June, with the north-northwest wind direction being the most common.

Field experiments were performed during the 2022 and 2023 seasons on 10-year-old Medjool date palms (*Phoenix dactylifera* L.) planted in sandy soil at 8 x 8 meters. Regular irrigation was applied twice every week in summer and once every week in winter, using 2 bubblers 180° (Model No. 8206B, Rain Bird, USA) (flow rate 120 l/h at a pressure of 1 bar) placed 50 cm away from the base of the stem. Each palm was surrounded by a drip circle of 16 mm tubing as the water source for the bubblers, which were installed on both sides of the stem. The 16 mm line was connected to sub-lateral lines that came from a sub-branch of the main irrigation network. Uniform date palms in terms of vigor, fruiting, and health were used in this experiment and received the same horticultural practices, i.e., irrigation program ~ 110−90 liters for each tree depending on the growth stage and evapotranspiration rate [18]. Fertigation applied essential elements nitrogen (N) 766.66 g., of phosphorus (P) 400 g., and potassium (K) 466.66 g. per palm [19]. Pruning was carried out to remove the earliest, latest, and smallest inflorescence, leaving 1 bunch/8 mature leaves to achieve the target of 10 fruit bunches per palm. Ghanami pollen grains were used for pollination as soon as the female spathes split in March, with direct covering afterward using paper bags to prevent pollen contamination and rain. To ensure even pollen distribution, bags were shaken, and the pollination process was repeated three times at 5-day intervals [20]. Pollination was performed using mechanical dusting between mid-March and the first week of April each year, applying 20 grams of fresh dry pollen per date palm tree mixed with an equal amount of wheat flour. The pollen used was collected in the same year from a male palm previously demonstrated to have excellent germination and fruit traits and was not from previous years to guarantee viability. The experimental design included six treatments starting at the end of November each year, applying the recommended amount of organic manure (50 kg), as is common practice in this area, as explained in Table 1 below:

**Table 1:** Description of study treatments.

Treatments	Description
T1 (Control)	Date palm trees grown in sandy soil without any addition of organic matter
T 2	Animal manure at the rate of 50 kg/ Palm
T 3	Poultry Manure at the rate of 50 kg/ Palm.
T 4	Filter mud at the rate of 50 kg/ Palm
T 5	25 kg animal manure + 25 kg filter mud/ Palm
T 6	25 kg poultry manure + 25 kg filter mud/ Palm

The organic matter was added at the end of December each year of the two experimental seasons to 30 palm trees, representing 5 palm trees for each treatment that were arranged in a complete randomized block design (CRBD).

### 2.2. Soil attributes and leave mineral content

Measurements and determinations included characteristics of orchard soils, organic matter used, irrigation water as per Tables 2, 3, 4 respectively. Analysis of soil was done as described by Bouyoucos [21]. The method of Grossman and Reinsch [22] was followed to estimate Bulk density (BD). For soil Texture samples of the physical analysis were taken from three layers 0–30 cm, 30–60 cm and 60–90 cm depth. In addition, electrolyte conductivity (EC), pH, organic matter (OM) and NPK were determined in soil samples from those layers, in filter mud, animal and poultry manure and in irrigation water the content of carbonate, bicarbonate, sodium and chloride were measured as well. The method of Micro-kjeldehl was used for total nitrogen determination as described by page [23].

**Table 2:** Soil characteristics of Medjool date palms at Bashandi Village, Dakhla Oasis, New Valley Governorate, Egypt.

Properties		Units	Soil depth (cm)		
			0–30	30–60	60–90
Particle size distribution	Fine sand	%	48.10	54.17	59.27
	Course sand	%	49.15	33.33	36.42
	Silt + Clay	%	2.75	12.50	4.31
pH (1:10)		–	8.45	8.51	8.83
EC (1:10)		ds m^-1^	4.77	4.99	5.55
OM		%	41	35	25
BD		g cm^-3^	1.65	1.60	1.55
N		%	0.18	0.15	0.11
P		%	0.15	0.10	0.07
K		%	0.17	0.13	0.10

Acidity (pH), Electrolyte Conductivity (EC), Organic Matter (O.M), N refers to Nitrogen, P refers to Phosphorus and K refers to Potassium. Bulk Density (BD).

**Table 3:** Irrigation water characteristics.

Properties	Units	Mean
pH (1:10)	–	8.10
EC (1:10)	ds m^-1^	1.42
CO3^2-^	Meq l^-1^	< 0.01
HCO^3-^	Meq l^-1^	0.11
Na^2+^	Meq l^-1^	1.89
Cl^-^	Meq l^-1^	0.50

Acidity (pH), Electrolyte Conductivity (EC), Carbonates (CO_3_), Bicarbonates (HCO_3_), Sodium (Na) and Chloride (Cl). The measurement unit was Meq/l.

**Table 4:** Characteristics of Filter Mud, Animal and Poultry Manure that used in this experiment.

Properties	Units	Filter Mud	Animal Manure	Poultry Manure
pH (1:10)	–	7.40	7.95	8.45
EC (1:10)	ds m^-1^	3.02	3.95	4.12
Total weight	m^3^	520	750	680
Humidity	%	60	65	63
OM	%	62.60	61.60	67.60
BD	g cm^-3^	0.95	1.25	1.18
Ash	%	37.90	33.90	33.90
N	%	1.95	1.85	2.15
P	%	2.79	0.71	0.79
K	%	0.15	0.85	1.85
C	%	34.90	30.70	35.90
C:N ratio	–	19:10	17:10	20:10

Acidity (pH), Electrolyte Conductivity (EC), Organic Matter (O.M), Bulk Density (BD), N refers to Nitrogen, P refers to Phosphorus and K refers to Potassium and C refers to Carbon.

The method of Cotteine et al., [24] was used for phosphorus determination. Potassium (K) and sodium (Na) were determined by flame photometer [25] and USDA [26]. Calcium concentration was determined using an atomic absorption spectrophotometer (Perkin Elmer-3300) [27], while magnesium (Mg) was measured according to Wilde et al., [28]. EC was determined as an indicator of ion leakage [29]. Based on the titration method of silver nitrate (0.025 M) with potassium chloride (0.l M), the chloride concentration was estimated [30]. The organic matter (OM) of the different composts used in this experiment was calculated using the method of AFNOR [31].

Organic carbon in farm soil was determined according Nelson and Sommers [32] and following this equation (1):


OM %=organic carbon (%) × 1.72 (1)

To follow moisture in the soil treatments, available water (AW) was calculated according to equation (2) of Klute [33]:


AW% =% WFC – % PWP  (2)

Where %WFC is the water content at field capacity at (−33kpa), %PWP is the water content at permanent wilting point at (−1500 kpa).

### 2.3. Yield parameters and fruit quality

Calculation of fruit set was done after three weeks of pollination via counting the number of fallen flowers, which was subtracted from the total number of flowers set according to the following equation (3):


$$\text{Fruit Set }\%=\frac{No.\;of\;Setting\;Flowers\;}{Total\;No.\;of\;Flowers}\text{ X}\text{1}\text{0}\text{0}$$ (3)

After counting the total numbers of flowers and the fallen flowers, the bunches were packed again. Paper bags were taken off over the bunches in the open air at the end of the biser stage (unripe stage). Then again, the number of dropped fruits was counted and subtracted from the total number of fruit set as per following equation (4):


$$\text{Fruit dropped }\%=\frac{No.\;of\;droped\;fruits\;}{No.\;of\;retention\;fruit\;+No.\;of\;droped\;fruits\;}\text{ X}\text{1}\text{0}\text{0}$$ (4)

In a small modification to Glasner [34], fruit harvest was done at first of the October each year between the Rutab and Tamr stages (fruit color in this stage was golden-brown, wrinkled plumper with high moisture content compared to the Tamr stage). After the harvest, fruit was brought to the laboratory and stored at room temperature (16^◦^C and 50–60% relative humidity) till measurements were done. The yield is normally calculated by dividing the average bunch weight (kg) by the number of bunches.

The physical and chemical properties of the fruits were determined by choosing fifty fruits randomly from each palm. Fruit height and diameter were measured using a digital vernier caliper. Sensitive balance (0.01 g) was used to determine total fruit weight, flesh weight, and seed weight, and to calculate the ratio of flesh to seed. Subsequently, for chemical property measurements, a mixture of 50 gram of fruit pulp with 50 ml of distilled water was prepared using a blender and allowed to stand. To determine the total soluble solids content (TSS %), a hand refractometer [35] was used. Following the volumetric method according to Lane and Eynon [36], the percentage of total and reducing sugar was measured. The non-reducing sugars were calculated as follows (equation 5):


Non−Reducing Sugars %=(Total Sugars−Reducing sugars) X100 (5)

The percentage of total acidity was estimated in terms of malic acid content using titration against 0.1M NaOH with phenolphthalein dye [35]. The percentage of crude fibers was measured using a mixture of acetic glacial solution and nitric acid at a10: 1 ratio [35].

### 2.4. Statistical analysis

The experiments were designed as plots with six treatments and five replicates representing 30 date palm trees in each year of the two experimental seasons and arranged in a completely randomized block design (CRBD). The recorded results were analyzed and normalized using Statistical Graphics Corporation, STATGRAPHICS Plus (St. Louis, MO, USA) with one-way analysis of variance (ANOVA). Duncan’s multiple range test was employed [37] at a significance level of 0.05 (L.S.D). Significant differences between treatments are indicated using different letters, while similar letters refer to no significance.

## 3. Results and discussions

### 3.1. Effect of filter mud, animal and poultry manure on water holding capacity in the soil

In general, the results of this experiment revealed that all types of compost used—i.e., filter mud, animal manure, and poultry manure—significantly increased all parameters related to the water-holding capacity of the experimental soil, including WFC, at PWP, AW, and the total content of NPK in comparison to the control (Table 5). It is interesting to note that in both seasons (2022 and 2023, respectively), filter mud alone (T4) performed noticeably better than animal manure (T2) or poultry manure (T3) in terms of storing water (WFC, AW). T4 represented almost12.1% of WFC and 7.5% of AW compared to 10.5% and 6.01% in T2, and 10.62% and 6.05% in T3, respectively, in both seasons. This illustrates how filter mud alone can retain moisture for extended periods. However, the treatment, combining filter mud with poultry manure (T6) demonstrated a significantly greater proportion of WFC and AW (12.59% and 8.09%, respectively) in both the seasons 2022 and 2023 seasons. In both seasons, the T5 treatment ranked second in terms of WFC and AW content, which were noticeably higher than those of the other treatments (T4, T3, and T2). Conversely, in terms of PWP, which showed a significant difference compared to the control (T1) in both seasons 2022 and 2023, there were no significant changes between any of the treatments. Similarly, the data in Table 5 show a considerable increase in soil enrichment measured as nutrients from T1 to T6 (i.e., total content of NPK); T6 exhibited the greatest results in that context.

**Table 5:** The effect of Filter Mud, Animal and Poultry Manure on WFC, PWP, AW and NPK during 2022 and 2023 seasons.

Variable	Units	Years	Treatments						SD	LSD (0.05)
			T1	T2	T3	T4	T5	T6		
WFC	%	2022	10.01E	10.53D	10.62D	12.10C	12.32B	12.58A	1.01	0.16
		2023	10.02E	10.55D	10.63D	12.12C	12.34B	12.59A	1.10	0.13
PWP	%	2022	4.21B	4.51A	4.57A	4.55A	4.56A	4.51A	0.14	0.13
		2023	4.23B	4.53A	4.59A	4.56A	4.57A	4.50A	0.14	0.10
AW	%	2022	5.80E	6.02D	6.05D	7.55C	7.75B	8.07A	1.02	0.13
		2023	5.79E	6.02D	6.04D	7.56C	7.77B	8.09A	1.04	0.12
N	mg. Kg^-1^	2022	30.35F	31.54E	31.75D	33.56C	34.12B	35.23A	1.84	0.08
		2023	30.23F	31.52E	31.76D	33.58C	34.15B	35.25A	1.10	0.13
P	mg. Kg^-1^	2022	3.41F	3.56E	3.89D	4.53C	4.85B	5.33A	0.62	0.12
		2023	3.42F	3.57E	3.92D	4.51C	4.87B	5.37A	0.77	0.12
K	mg. Kg^-1^	2022	56.32F	57.53E	58.11D	59.58C	60.32B	61.21A	1.84	0.120
		2023	56.33F	57.51E	58.13D	59.55C	60.33B	61.18A	1.83	0.16

T1: untreated soil (control), T2: animal manure at 50 kg/ palm, T3: poultry manure at 50 kg/ palm, T4: filter mud at 50 kg/ palm, T5: filter mud at 25 kg/ palm + animal manure at 25 kg/ palm, T6: filter mud at 25 kg/ palm + poultry manure at 25 kg/ palm. Water content at field capacity (WFC), Water content at permanent wilting point (PWP), Available water (AW), N refers to Nitrogen, P refers to Phosphorus and K refers to Potassium. Means followed by the same letter are not statistically different by Duncan at 0.05 levels. SD refers to stander deviation.

Our findings were consistent with those of [38,39], who observed that the addition of organic manures to the soil of the Barhy date palm cultivar enhanced the soil’s ability to retain water and nutrients, particularly NPK. Additionally, Elsadig et al., [40] found that applying organic manure along with regular NPK treatments enhanced the soil’s ability to hold nutrients and water content parameters in the Khenazi date palm cultivar (*Phoenix dactylifera* L.). Similarly, Noufal [8] demonstrated that applying filter mud improved the characteristics of desertic soil in terms of retaining nutrients and water. Accordingly, Abo-Ogiala and Khalafallah [41] and Aly and Zagzog [42] showed that the cultivation of organic matter enhanced the soil’s ability to retain water and nutrients in the farms of Zaghloul date palm and Washington navel orange, respectively. Also, Ou-Zine et al., [43] found that organic amendment increased soil capacity of the majhoul date palm cultivar in Drâa-tafilalet region, Morocco. In addition, El Janati et. al., [13] showed similar results in that work, where they stated that soil structure reflected an increase in holding capacity for water and nutrients due to the application of organic matter, especially date palm residues compost.

Furthermore, Morales-Maza et. al., [16] found that application of compost beside chemical fertilization in the farm of Medjool date palm increased soil capacity in holding nutrients and water.

### 3.2. Effect of filter mud, animal and poultry manure on leave mineral content

The data in Table 6 show the mineral content of Medjool date palm resulting from the applied treatments in the experiment. As shown in Table 6, the treatment with filter mud alone (T4) resulted in significantly lower levels of N and P (8.31 and 1.77 mg. g^-1^) compared to T3 (8.55 and 1.88 mg. g^-1^), T5 (9.55 and 2.15 mg. g^-1^) and T6 (9.88 and 2.43 mg. g^-1^) and significantly higher levels compared to T1 (6.55 and 1.13 mg. g^-1^) and T2 (7.54 and 1.75 mg. g^-1^), respectively, in the 2023 season, which was similar to 2022. Concerning K^+^, Ca^+2^, and Mg^+2^ concentrations, the data showed a significant ascending increase from T1 to T6 in both the 2022 and 2023 seasons. However, T3 did not differ significantly in K concentration from T4 in the 2022 season. These findings are in agreement with [8,15], who reported that the highest available N, P, K^+^, Ca^+2^, and Mg^+2^ content was obtained with the highest application rate of the organic amendment, likely due to the increase in microbial biomass [44]. Their interpretation was that application of organic manure increased cation exchange capacity (CEC), i.e., the total and available amount of these nutrients, and subsequently their uptake. Conversely, the data showed a significant decrease in Na and Cl content from T1-T6 in both seasons 2022 and 2023, respectively. This suggests that organic manure in all treatments improved soil structure by retaining Na^+^ and Cl^-^ ions and promoting their accumulation in roots, which was reflected in their decreased content in leaves. These findings align with those of [45], who reported that some plant species exclude salt ions, especially Na^+^, Cl^-^, via gland cells—a mechanism absent in date palms. Therefore, a key adaptation to salinity in many date palm varieties is the compartmentalization Na^+^, Cl^-^ ions in roots, preventing their translocation to the leaves [46–48]. According to the same pattern, the application of organic fertilizer boosted the mineral content of the leaves of the Zaghloul date palm and Washington navel orange, respectively [41,42]. Applying filter mud lowers the cost of chemical fertilizer by 15% to 20% in various agricultural crops, which is quite close to our goal [5]. According to Morales-Maza, et al., [16]. The Medjool date palm farm’s soil is evidently enriched with nutrients by the addition of compost as organic manure in addition to chemical fertilization.

**Table 6:** The effect of Filter Mud, Animal and Poultry Manure on N, P, K, Ca, Mg, Na, and Cl during 2022and 2023 seasons.

Variable	Units	Years	Treatments						SD	LSD (0.05)
			T1	T2	T3	T4	T5	T6		
N	mg. g^-1^	2022	6.55F	7.54E	8.55C	8.21D	9.55B	9.87A	1.24	0.14
		2023	6.57F	7.51E	8.51C	8.31D	9.49B	9.88A	1.22	0.10
P	mg. g^-1^	2022	1.15E	1.74D	1.88C	1.77D	2.13B	2.41A	0.42	0.04
		2023	1.13E	1.75D	1.87C	1.76D	2.15B	2.43A	0.44	0.05
K	mg. g^-1^	2022	5.11E	6.78D	7.85C	7.89C	8.75B	9.16A	1.47	0.06
		2023	5.13F	6.77E	7.84D	7.91C	8.77B	9.15A	1.46	0.05
Ca	mg. g^-1^	2022	2.41F	3.44E	3.55D	3.65C	4.56B	4.89A	0.88	0.05
		2023	2.43F	3.41E	3.56D	3.64C	4.57B	4.87A	0.87	0.05
Mg	mg. g^-1^	2022	1.11F	1.43E	1.75D	1.88C	2.13B	2.51A	0.50	0.05
		2023	1.14F	1.47Ee	1.77D	1.89C	2.14B	2.49A	0.48	0.06
Na	mg. g^-1^	2022	7.91A	6.55B	5.99C	6.12D	5.11E	5.12E	1.04	0.07
		2023	7.88A	6.57B	5.93C	6.07D	5.15E	5.09E	1.03	0.07
Cl	mg. g^-1^	2022	17.98A	15.56B	14.88C	15.14D	14.13E	14.11E	1.43	0.10
		2023	17.87A	15.66B	14.78C	15.11D	14.17E	14.15E	1.40	0.07

Same footnotes of Table 4: plus, Ca refers to Calcium, Mg refers to Magnesium, Na refers to Sodium and Cl refers to Chloride. SD refers to stander deviation.

### 3.3. Effect of filter mud, animal and poultry manure on yield parameters

**Fig 2:**
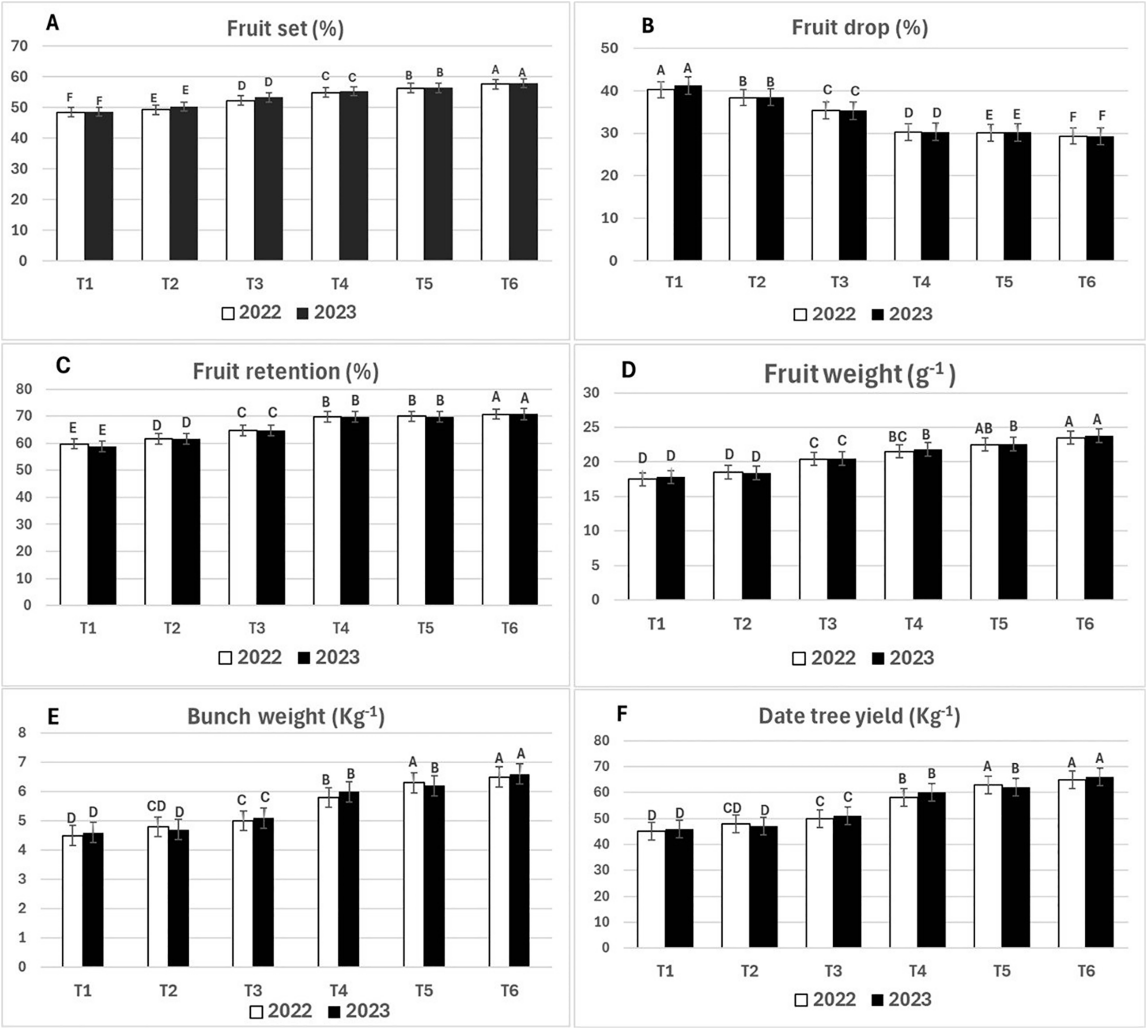
The effect of Filter Mud, Animal and Poultry Manure on fruit set, fruit drop, fruit retention, fruit weight, bunch weight and date tree yield during 2022 and 2023 seasons. T1: untreated soil (control), T2: animal manure at 50 kg/ palm, T3: poultry manure at 50 kg/ palm, T4: filter mud at 50 kg/ palm, T5: filter mud at 25 kg/ palm + animal manure at 25 kg/ palm, T6: filter mud at 25 kg/ palm + poultry manure at 25 kg/ palm. Means followed by the same letter are not statistically different by Duncan at 0.05 levels. Error bars represent standard errors.

The data in Fig 2 (A) show a significant ascending increase in the percentage of fruit set from T1 to T6 (48.57, 50.25, 53.26, 55.24, 56.36, and 57.85, respectively, in the 2023 season). These are like those observed in 2022, reflecting the positive effect of filter mud, animal manure, and poultry manure and their mixtures on fruit set in both experimental seasons (2022 and 2023). On the other hand, the results in Fig 2 (B) demonstrate a decreasing impact on fruit drop from T1 to T6. Regarding fruit drop, T4 and T5 did not differ significantly, whereas the other treatments showed notable differences (41.21, 38.47, 35.31, 30.28, 30.21, and 29.25, respectively), in the 2023 season, which closely matched data found in 2022. Additionally, the application of filter mud, animal manure, and poultry manure progressively increased fruit retention, fruit weight, bunch weight, and date palm yield from T5 to T6 compared to the control (T1) as shown in Fig 2 (C, D, E, and F). Regarding fruit retention, fruit weight, bunch weight, and date tree yield, there were no significant differences between T4 and T5, but the data indicated significant differences among the other treatments except between T1 and T2, which showed no significant differences in terms of fruit weight, bunch weight, and date palm yield, as illustrated in Fig 2 (C, D, E, and F). These findings demonstrated that filter cake mud, alone and without animal or poultry manure, can produce favorable results. However, the combined application of filter mud with poultry manure (T6) yielded the best outcomes compared to the other treatments. It is possible that increasing the application rate of filter mud alone produce comparable effects, which warrants further investigation in future studies. Our results align with previous research by Marzouk and Kassem [49], Elsadig [40], and El-Sayed et al., [50], which examined how application of organic matter, either alone or in combination with mineral fertilizers, can enhance yield characteristics. In depth, the yield of Zaghloul dates increased significantly by 8–17% when organic matter was applied in combination with chemical fertilizers, compared to the use of chemical fertilizers alone [49]. This reflects decreased fruit drop and an increase in fruit set, fruit retention, fruit weight, and bunch weight. In addition, El-Sayed et al., [50] found that the yield of Barhy date palms cultivated in Upper Egypt increased 13% upon application of organic manure mixed with mineral fertilizers in comparison with chemical fertilizer alone. In the same trend [16,43], who reported that organic amendment showed a clearly rising yield of Medjool date palm cultivar to the different levels ranging from 19% to 49%. Difference in yield production return to differences in date palm age, cultivars, climate, soils, sources and doses of organic or chemical fertilizers.

### 3.4. Effect of filter mud, animal and poultry manure on fruit quality

**Fig 3:**
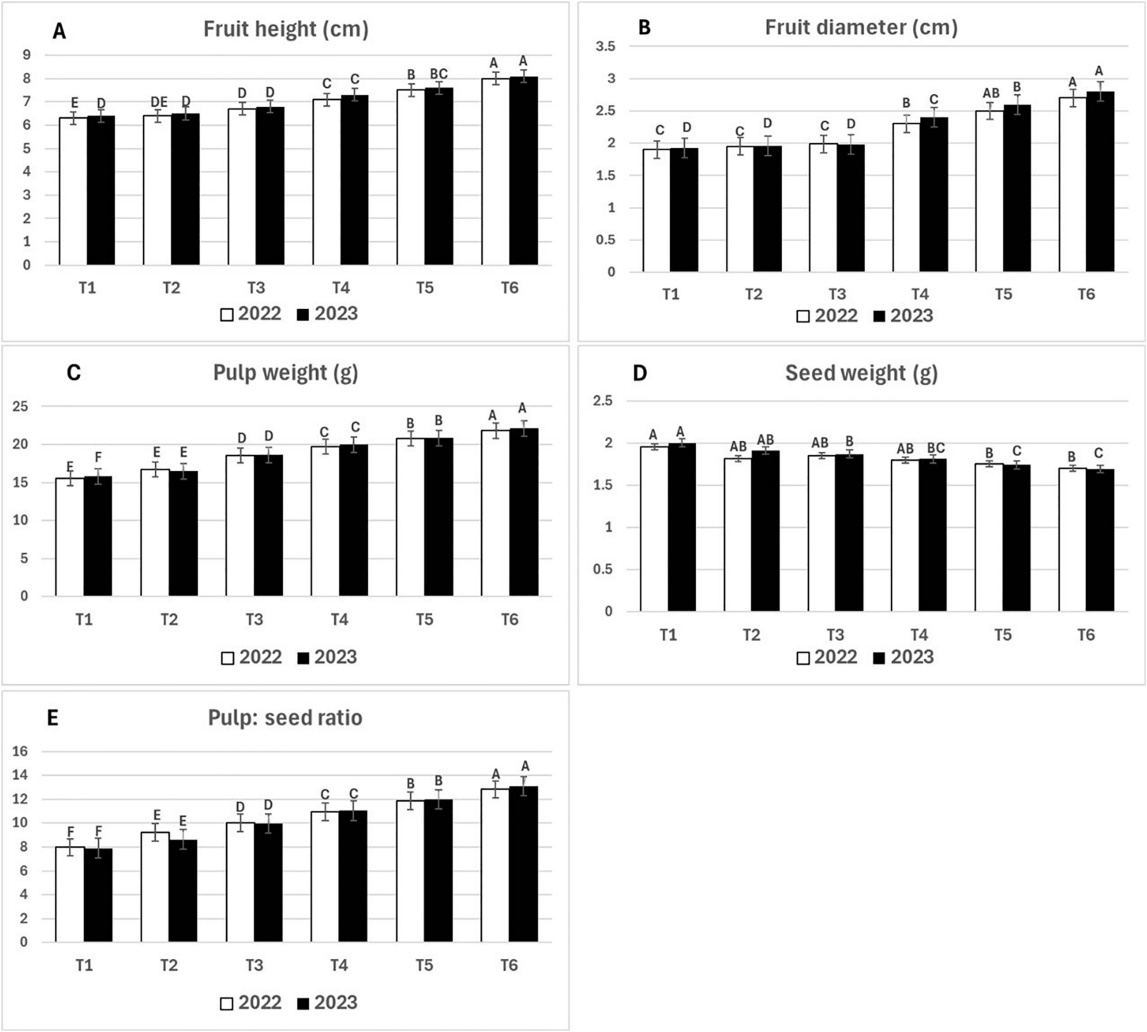
The effect of Filter Mud, Animal and Poultry Manure on fruit height, fruit diameter, pulp weight, seed weight and pulp: seed ratio during 2022 and 2023 seasons. T1: untreated soil (control), T2: animal manure at 50 kg/ palm, T3: poultry manure at 50 kg/ palm, T4: filter mud at 50 kg/ palm, T5: filter mud at 25 kg/ palm + animal manure at 25 kg/ palm, T6: filter mud at 25 kg/ palm + poultry manure at 25 kg/ palm. Means followed by the same letter are not statistically different by Duncan at 0.05 levels. Error bars represent standard errors.

Data in Fig 3 (A, B, C, D, and E) showed that T6 treatment produced significantly superior results for fruit height (cm), fruit diameter (cm), pulp weight (g), seed weight (g), and pulp-to- seed ratio (8.1, 2.8, 22.11, 1.69, and 13.08, respectively) compared to the other treatments in the 2023 season. These findings follow a similar trend to those observed in 2022. However, T2 showed better results than T1, but there were no significant differences between them in all previously mentioned parameters, except for pulp weight and pulp- to-seed ratio in both the 2022 and 2023 seasons. In other side, T3 showed better results compared to T1 and T2 in all parameters of fruit quality but there were no significant differences between T3, T2, and T1 concerning fruit height and fruit diameter in both seasons, 2022 and 2023, respectively. Furthermore, T4 and T5 showed significantly superior results compared to T1, T2, and T3 in the measured fruit quality parameters except for seed weight, where no significant difference was observed between T4 and T3 in both seasons. On the other hand, T5 yielded better results than T4, but there were no significant differences between them in measured parameters of fruit quality parameters in both seasons, 2022 and 2023, respectively, as shown in Fig 3 (A, B, C, D, and E).

On the other side, data in Table 7 showed ascending percentage increase in TSS, total sugars, reducing and non-reducing sugars from T1 till T6 where T6 showed the best results for the mentioned parameters (67.1, 66.8, 14.11 and 52.69, respectively), while showed percentage descending in total acidity, total soluble tannins, and total crud fibers where T6 showed the lowest data for those parameters (0.236, 0.39 and 0.70, respectively) in 2023 season which showed similar data directions that found in 2022. Moreover, T6 showed significantly superior data compared to T1, T2, T3, and T4 in all mentioned quality metrics except total sugars, but not significantly compared to T5.

**Table 7:** The effect of Filter Mud, Animal and Poultry Manure on total soluble solids contents (TSS), total sugars, reducing and non-reducing sugars, total acidity, total soluble tannins, and total crud fibers during 2022 and 2023 seasons.

Variable	Units	Years	Treatments						SD	LSD (0.05)
			T1	T2	T3	T4	T5	T6		
TSS	%	2022	61.30D	63.41C	64.70C	65.11BC	66.50AB	67.01A	1.00	1.75
		2023	61.41D	63.50C	64.80B	65.31B	66.60A	67.11A	2.10	0.87
Total sugars	%	2022	60.90C	61.91C	62.96BC	64.05ABC	65.51AB	66.70A	2.19	3.34
		2023	60.93C	61.95C	62.98BC	64.15ABC	65.61AB	66.80A	2.22	3.59
Reducing sugars	%	2022	11.50E	11.69E	12.55D	13.01C	13.55B	14.01A	1.00	0.32
		2023	11.51E	11.59E	12.63D	13.19C	13.56B	14.11A	1.058	0.26
Non-reducing sugars	%	2022	49.4D	50.22CD	50.41CD	51.04BC	51.95AB	52.69A	1.21	1.27
		2023	49.42C	50.36B	50.35B	50.96B	52.04A	52.69A	1.21	0.91
Total acidity	%	2022	0.25A	0.25AB	0.25AB	0.25BC	0.24CD	0.24D	0.01	0.08
		2023	0.26A	0.25A	0.24AB	0.24BC	0.23CD	0.23D	0.01	0.01
Total soluble tannins	%	2022	0.55A	0.53A	0.49B	0.44C	0.41D	0.40D	0.06	0.03
		2023	0.57A	0.52B	0.48C	0.43D	0.42D	0.39E	0.07	0.03
Total crud fibers	%	2022	1.21A	1.19A	0.88B	0.80C	0.75CD	0.71D	0.22	0.07
		2023	1.22A	1.20A	0.85B	0.82B	0.74C	0.70C	0.23	0.07

T1: untreated soil (control), T2: animal manure at 50 kg/ palm, T3: poultry manure at 50 kg/ palm, T4: filter mud at 50 kg/ palm, T5: filter mud at 25 kg/ palm + animal manure at 25 kg/ palm, T6: filter mud at 25 kg/ palm + poultry manure at 25 kg/ palm. TSS refer to total soluble sugars. Means followed by the same letter are not statistically different by Duncan at 0.05 levels.

On the other hand, T1 and T2 did not differ significantly in any of the quality metrics that were mentioned, but they did differ significantly from T3 in terms of TSS, reducing sugars, total soluble tannins, and total crude fibers. Additionally, Table 7 indicates that T3 and T4 did not differ significantly in any of the mentioned quality indicators except reducing sugars and total soluble tannins. Similar results were found between T4 and T5, where there were no significant differences between them except in the reducing sugars parameter. The results in both season 2022 and 2023, showed differences between them concerning significance between treatments in the in all referred quality parameters, as precisely described in Table 7. These results supported those found by Marzouk and Kassem [49] on Zaghloul dates and by Al-Kahtani, and Soliman [38] on Barhy dates, where they explored the positive effects of organic manures on improving the same parameters of fruit quality as those measured in this study. In addition, Elsadig et. al., [40] found that application of organic manure with normal rates of NPK fertilization clearly increased the parameters of fruit quality of the Khenazi date palm cultivar. Furthermore, El-Sayed et al., [50] explored the enhancement of fruit parameters of Barhy date palms cultivated in Upper Egypt due to the application of organic matter alone or combined with mineral fertilizers. Furthermore, Ou-Zine et. al. [43], and Morales-Maza et al., [16], reported that organic amendment showed significant superiority in fruit parameter of the Medjool date palm cultivar in comparison to control.

## 4. Conclusion

This study strongly aligns with SDG targets related to achieving sustainable food production. The substantial residue left after sugarcane harvest contributes to negative environmental impacts. Recycling this residue yields various organic matter products that serve as sustainable substrates for agricultural production. Filter mud, as one such product, has been previously tested as a soil amendment, demonstrating promising benefits for soil physicochemical and microbial properties compared to common alternatives like animal and poultry manure.

Furthermore, in this study, the application of filter mud—alone or mixed with animal or poultry manure—showed significantly better results for all parameters investigated (water-holding capacity, leaf mineral content, yield, and fruit quality of Medjool date palm). Specifically, the application of filter mud mixed with poultry manure (T6) produced the best results across all parameters, followed in descending order by the other treatments (T5, T4, T3, T2, and T1, respectively). Due to the limitation of applying a higher dose of filter mud in this study—necessitated by the need for comparison with common manure in the field and to avoid challenges related to transportation, cost, and extensive field and laboratory work—the authors suggest further studies with higher doses of filter mud alone, instead of mixing it with other manures. Additional measurements, especially of microelements, are also recommended.
